# “Thinking on your feet”: A qualitative evaluation of sit-stand desks in an Australian workplace

**DOI:** 10.1186/1471-2458-13-365

**Published:** 2013-04-18

**Authors:** Anne Carolyn Grunseit, Josephine Yuk-Yin Chau, Hidde Pieter van der Ploeg, Adrian Bauman

**Affiliations:** 1 Prevention Research Collaboration, School of Public Health, University of Sydney, Level 2, Medical Foundation Building K25, 92 Parramatta Rd, Camperdown, NSW, 2006, Australia; 2 Department of Public and Occupational Health, VU University Medical Center, van der Boechorststraat 7, Amsterdam, 1081BT, Netherlands

## Abstract

**Background:**

Epidemiological research has established sitting as a new risk factor for the development of non-communicable chronic disease. Sit-stand desks have been proposed as one strategy to reduce occupational sedentary time. This formative research study evaluated the acceptability and usability of manually and electrically operated sit-stand desks in a medium-sized government organisation located in Sydney, Australia.

**Methods:**

Sitting time pre- and three months post -installation of the sit-stand desks was measured using validated self-report measures. Additionally, three group interviews and one key-informant interview were conducted with staff regarding perceptions about ease of, and barriers to, use and satisfaction with the sit-stand desks. All interviews were recorded, transcribed and analysed for themes regarding usability and acceptability.

**Results:**

Of 31 staff, 18 completed baseline questionnaires, and 13 completed follow-up questionnaires. The median proportion of sitting time for work was 85% (range 50%-95%) at baseline and 60% (range 10%-95%) at follow-up. Formal statistical testing of paired data (n=11) showed that the change from baseline to follow-up in time spent sitting (mean change=1.7 hours, p=.014) was statistically significant. From the qualitative data, reasons given for initiating use of the desks in the standing position were the potential health benefits, or a willingness to experiment or through external prompting. Factors influencing continued use included: concern for, and experience of, short and long term health impacts; perceived productivity whilst sitting and standing; practical accommodation of transitions between sitting and standing; electric or manual operation height adjustment. Several trajectories in patterns of initiation and continued use were identified that centered on the source and timing of commitment to using the desk in the standing position.

**Conclusions:**

Sit-stand desks had high usability and acceptability and reduced sitting time at work. Use could be promoted by emphasizing the health benefits, providing guidance on appropriate set-up and normalizing standing for work-related tasks.

## Background

There is growing evidence that sitting and sedentary behaviour are negatively associated with health outcomes independently of physical activity [1,2]. Research shows that prolonged occupational sitting has acute negative metabolic effects [3] as well as associations with greater cardiovascular morbidity, weight gain [4] and premature death [5,6]. Given among developed countries the high proportion of the adult population that is employed (e.g., over 70% of persons aged 15–64 in Australia) [7], that workers spend over one-third of their waking hours at work [8], and the trend for modern occupations is towards sedentary tasks (e.g., the prevalence of moderate intensity physical activity occupations decreased from 48% in 1960 to 20% in 2008) [9] the contribution of occupational sedentary behaviour to the public health burden is likely to be considerable. Conversely, reducing and/or breaking up sitting time at work may potentially substantially attenuate the risk of metabolic and cardiovascular disease amongst the working [3,10].

Thus current epidemiological knowledge warrants targeting sitting as the primary outcome for future workplace interventions and evaluations [11]. Sit-stand desks have been proposed as one such strategy to reduce occupational sedentary time [12]. Sit stand desks are desks or work surfaces retrofitted to standard desks that allow height adjustment such that the user may work comfortably either sitting or standing. The height-adjustable surface may be the entire desk or an attachment that raises and lowers a computer screen, keyboard and mouse.

There is evidence that installing sit-stand desks in a workplace can result in significantly reduced sitting time at work [13,14]. Further, with one exception [15], evaluations of physical impacts of sit-stand desks have found lower rates of physical discomfort/complaints compared with sitting [16-19] or standing [20] control conditions and are not associated with decreases in objectively measured productivity (efficiency and accuracy) [15,16,18]. Assessments of usability and acceptability have been primarily quantitative and have shown positive employee reactions [13,14,17,21]. Wilks, Mortimer and Nylén (2006) conducted interviews with desk purchasers on the process of obtaining and implementing the desks and reported interviewees felt the investment to be a success despite low utilisation. Unfortunately few insights could be gained explaining low usage as employees were not interviewed about using the desks [21]. An Australian study used qualitative methods to investigate strategies for reducing occupational sitting time amongst office workers, with sit-stand desk use as one alternative discussed [12]. However, the discussion was based on participants projected opinions rather than actual experience. The study reported here adds to the body of evidence on the impact of sit-stand desks by collecting and analysing detailed, qualitative information about user experience in conjunction with measuring changes in sitting time grounded in a real world setting.

Therefore, the aim of the current study was to conduct formative research examining the impact, acceptability and usability of sit-stand desks among office workers using mixed methods. Specifically, we wanted to describe not only whether the sit-stand work stations reduced employees’ sitting time, but also the factors employees reported affecting their initiation and maintenance of use of the desks in the standing position.

## Method

### Study setting and design

The study setting was a medium sized [22] government organisation providing leadership and management training in Sydney, Australia. The evaluation was independent and opportunistic (rather than planned) as the approach to the organisation to conduct the research occurred subsequent to the decision to procure and install the sit-stand desks. Sit-stand desks were installed in early November 2011 as part of a major office refurbishment. Staff were relocated to another building within the same worksite whilst works were undertaken and then relocated back to the refurbished premises. All staff were allocated manual (wind-up, Schiavello Centric range, $AUD1090) or electric (Schiavello Centric range $AUD1345) sit-stand desks or in some cases one of each. Because of cost, all offices but only 10% of the open plan area (randomly allocated) had electrically operated desks. The desks themselves were rectangular (2100 mm × 800 mm) or corner-desk and the entire work surface was height adjustable.

The evaluation was primarily qualitative consisting of group interviews and one key-informant interview with staff. Quantitative data was also collected on self-reported sitting for descriptive purposes and for recording demographic characteristics.

### Procedure

The researchers visited the organisation premises and addressed an all-staff meeting to apprise staff of the evaluation and leave information and consent forms. While the information session encouraged staff to take part in the study, they were neither encouraged nor discouraged to use the desks, nor was any information regarding the potential health benefits of standing conveyed. Subsequently, an email invitation to participate was forwarded to staff and 31 (of 33 total staff) employees were onsite and invited to participate. Non-responders were sent two email reminders. Staff interested in participating in the evaluation were asked to read the participant information and complete consent forms and return them by email to the researchers.

Consenting staff were sent baseline questionnaires (a modifiable pdf) prior to moving to the new premises which were returned by email. Approximately four months later (three months post-installation), emails were sent asking those who took part at baseline if they would participate in group interviews concerning their experience with the desks and complete a second survey. Group interviews and the one key informant interview were conducted during working hours at the worksite. At the beginning of the interviews, follow-up questionnaires were distributed and completed by participants.

The research was approved by the University of Sydney Human Research Ethics committee.

### Quantitative survey instrument

The survey obtained demographic characteristics, height and weight, and workday sitting time and total sitting time. The Occupational Sitting and Physical Activity Questionnaire (OSPAQ) [23] measures the self-reported proportion of time spent sitting, standing, walking and doing more physically demanding tasks at work on a typical day in the last seven days. The Workforce Sitting Questionnaire (WSQ) [24] for domain-specific and total sitting time in working adults gathers self-reported sitting time in different domains (e.g., at work, for transport, using the computer) by work and non-workdays. All measures were retrospective.

The follow-up questionnaire included additional questions identifying whether the participant had received an electric or manual desk and work-related tasks (phone calls, emailing, reading etc.) done sitting and/or standing.

### Qualitative data collection

#### Key informant interview

The staff member responsible for sourcing and purchasing the sit-stand desks was interviewed regarding the acquisition process including inception, approval, procurement and installation.

#### Group interviews

The group interview guide was developed by the researchers who have expertise in sedentary behaviour research (HvP, JC, AG) and qualitative methods (AG). The guide consisted of a number of areas for discussion that directly addressed the aims of the study. Initial questions sought information on participants’ occupational roles, background with the organisation and the extent to which they work at a desk. Discussion then followed a temporal trajectory seeking information on means of, and reaction to hearing about the sit-stand installation; initiation of use; descriptions of their current use and why and how that may have changed since the desks’ installation. Within this broad framework, we probed for influences on the decision whether to sit or stand, how the desk affected participants’ work, and any effects on physical activity and sitting outside the workplace. Discussions were lead by the researchers (AG and JC) and followed a semi-structured format. The key informant interview and group interviews were recorded with the consent of participants and transcribed verbatim.

### Analysis

The qualitative data were analysed with a view to gaining a contextualised understanding the factors affecting initiation and maintenance of use of the height adjustable function of the desks. Themes relevant to these aims were generated from the content of the interviews rather than formulated apriori consistent with a grounded theory approach [25]. In the first stage of analysis, segments of text were tagged where interviewees described initiation and maintenance of use of the desk along with contextual data on prior expectations, extent of use, tasks carried out in the sitting or standing position and perceived impact. In the second stage, data were retrieved and reanalyzed for recurrent themes through an iterative process whereby commonalities in explanations of patterns of use were identified along with exceptions and then tested against the data. The data were analysed using QSR NVivo version 9.2 [26] by the lead researcher (AG) and cross-checked with another researcher (JC). JC reviewed the coding and any discrepancies were resolved by discussion. Interpretations were refined in consultation with the other authors.

Quantitative data were analysed using Stata version 11.1 [27]. Paired t-tests (one-tailed) and Wilcoxon matched pairs signed ranks tests were used to examine reduction from baseline to follow-up in time spent and proportion of time spent sitting at work for paired data.

## Results

### Sampling and participants

Eighteen of 31 staff members completed baseline questionnaires between six weeks and three days prior to relocation to the new premises (one further person consented but did not participate due to work demands). We have no further information about those who did not participate except that all received a sit-stand desk. Of those who completed a baseline survey, 11 completed a follow-up questionnaire, three left the organisation or were on extended leave, two expressly requested to be withdrawn from the study, one failed to respond regarding the follow-up and one person’s desk was broken from the outset. Two staff members who were unavailable at baseline (one was overseas and the other started with the organisation two months after installation) subsequently joined the study and completed follow-up questionnaires and participated in a group interview.

Of those for whom baseline and/or follow-up data were available (n=19^a^) 53% were female (n=10), were aged between 27 and 59 (median 46 years), almost 80% had a tertiary education (n=15) and all except one worked full-time, the median hours worked being 41 per week. All participants had desk-based jobs and were in a mix of administrative support, teaching (approximately one-third face-to-face, two-thirds desk-based preparation) and research roles.

### Quantitative data

Median proportion of the day spent sitting at baseline (n=17) was 85% (range 50% to 95%), and the mean self-reported sitting time for work was 6.9 hours (SD =1.2, range 5 to 8.5 hours). At follow-up (n=13), 92 days post installation (mean time between baseline and follow-up survey 122 days), the median proportion of time spent sitting at work was 60% (range 10% to 95%) with hours spent sitting averaging 5.4 (SD = 2.3, range 2 to 8.5 hours). Formal statistical testing of paired data (n=11) showed that the reductions from baseline to follow-up in proportion of time spent sitting at work (mean change=23% (95%CI: 4% to 41%), p=.011) and time spent sitting (mean change=1.7 hours (95%CI: 14 minutes to 3.2 hours), p=.014) were statistically significant (confirmed by non-parametric tests).

The proportions of the sample reporting at follow-up that they emailed (62% vs. 69%) and read (69% vs. 62%) sitting and/or standing respectively were similar. Differences in tasks performed in the sitting or standing position (not formally tested) were more apparent for meetings (77% vs 39%), writing (69% vs 46%) and phone calls (69% vs 54%) respectively.

### Qualitative data

The three group interviews of four people per group lasted between 39 and 52 minutes. Of those who attended, six had a manual desk or desks only, two had an electric desk only, and four had one of each type of desk (as did the key informant). Three group participants had tried the desk in the standing position but were infrequent users (less than daily) [21], one was a regular (daily) user but had sustained an unrelated lower limb injury and consequently had stopped standing, one participant had not used his desk in the standing position at all and the remainder (n=8) were regular (daily) users.

Staff awareness about the impending introduction of the desks varied, with a number saying they found out through informal channels or as a by-product of being informed about the evaluation study. Prior conceptions of the desks included “a luxury”, “interesting” and “innovative” and one participant conflated the concept with hot desks (a shared desk available to any employee). Many had not encountered the concept before.

In the following analysis, discussion about how the desks were used is framed around initiation of use, maintenance of use and the effect of manual versus electric height adjustment.

### Initiation

According to the participants (although the key informant reported otherwise) there was no formal instruction about how to set up a workspace to accommodate the height-adjustable desk. Initiation of use therefore had no systematic or formal prompt.

For some employees, the idea to use the standing option was lost in the myriad of other changes and tasks that accompanied the refurbishment. Explanations behind first use of the standing option could be classified into two main themes: 1) an anticipated health benefit, and 2) experimentation with no particular expectations or because of external prompting.

### Health-driven initiation

Motivated by the potential health benefits, six people appeared to commit to using the desks prior to installation:

I think I must have had a back ache at the time or something and I thought it sounded like a really great idea to be able to spend some time in the day standing rather than sitting the whole day.

Participant 11 (Group 3), manual desk, support/administrative role

I saw the articles, read the articles, talking about the amount of energy you exert standing as opposed [to sitting] and some of the sort of health benefits so I was quite excited about this thing.

Participant 18 (Group 2), electric & manual desks, teacher/manager role

According to their own accounts, those who reported to be motivated prior to installation for health reasons were also “early adopters”, and continued to be committed and enthusiastic users. One employee who had used sit-stand desks in a number of previous positions described his initiation elsewhere in such terms:

I got really interested with a small group of colleagues, middle aged men sitting around talking about our dodgy backs. And one of the guys had quite a serious injury and was recently returned from surgery and he had invested in one of these to help out with his recovery and to keep him at work. And, so I thought well I might have a look and I went around now I found some really cheap ones, so I got one to give it a go and really haven’t looked back. Within probably three months, I went out and purchased a whole stack more of them; wrangled some money out of our, found some money, and I got one and put them in each of the work units in my division.

Participant 20 (Group 2), manual desk, researcher role

Thus the potential health effects of providing an option for standing were a strong motivator for some employees.

#### Experimentation or external prompting

Among other staff members, raising their desk to standing height was described more as experimentation rather than as part of a health strategy. One employee trialed her desk because of the evaluation; others were prompted to use the stand option directly or indirectly by other staff in their work area using it:

I wasn’t using it, but I’ve got a bit of a bad back and bit of a shoulder injury as well. So, for some reason it just never occurred to me to actually wind it up and a colleague said to me you should give it a go, so I did.

Participant 1 (Group 2), manual & electric desks, support/administration role

Oh the two of us in the office that use it, like I started using it and then M started, she started using hers and now we use them quite regularly where the other two, N went ‘til her desk broke… R has never, she’s not even interested in trying so yeah but N seen us two using it and she’s gone ‘I might try it too’.

Participant 9 (Group 3), manual desk, support/administrative role

Without a specific motivation, however, widespread initiation appears to be left to happenstance and one participant believed this may lead to lower uptake across the workplace:

So in the first instance it’s a personal individual thing but if someone becomes enthusiastic about it and receives the benefits then it’s quite likely that that will impact other people in the workplace. Conversely, if no one in the workplace is trying it, it takes an individual to stand up to try it and if everyone else is not then that may also impact.

Participant 16 (Group 1), electric desk, teacher/manager role

### Maintenance

A number of factors emerged strongly in discussion of maintenance of use of the standing option of the desks. These could be summarized as: health/physical impacts; productivity/mental impacts, office set-up/context.

#### Health/physical impacts

As with the initiation phase, health or physical impacts were a reason people gave as to why they continued to use the desk in the standing position. Impacts could be physical as with the following staff member:

Today I’ve got it up and I’ve a bad back anyway so it’s good you know to actually, when you stand it gets a bit easier.

Participant 11 (Group 3), manual desk, support/administrative role

Health impacts were also expressed in terms of energy as described by this employee in response to a question regarding whether standing at work reduced other physical activity:

I still have the same level of activeness, if not probably more. Actually I still feel pretty energised when I get home.

Participant 1 (Group 2), manual & electric desks, support/administration role

Similarly, a couple of users mentioned not only standing but moving more with a raised desk.

And I do tend to move around my office a lot more as well. So I will go over and get something or I’ll walk out where as if you have to get up and walk away from your chair. I’m probably less likely to do it I’ll save it as a group of things so I’ll only have to go down once.

Participant 1 (Group 2), manual & electric desks, support/administration role

…one of the other things … which may be significant is I use the voice recognition software and I find that I can pace or walk and talk like I can think about what I’m saying.

Participant 18 (Group 2), electric & manual desks, teacher/manager role

Others would need to put the desk down if tired, although this did not completely foreclose on using the desk but rather limited the time in the standing position.

I wish I could do it longer, I’d like to do it for longer but by about yeah, after about an hour and half oh I think I need to sit down.

Participant 13 (Group 3), electric & manual desks, support/administration role

For one employee, despite a willingness to use the desk, the exacerbation of a physical complaint precluded continued use of the standing option:

I can move sometimes with difficulty but standing in one spot is putting more pressure on my back, and automatically start after a while it just shoots pain down the legs… There is nothing wrong with the desk, it’s me.

Participant 12 (Group 1), electric desk, support/administration role

Hence, physical impacts affected people’s use of the standing option both positively and negatively.

#### Perceived work productivity/mental impacts

A second factor reported to affect maintaining use of the height adjustment was the degree to which the potential user felt the standing position assisted or not with their productivity. For example, the following quotes illustrate where employees felt more efficient and/or alert when standing:

In that in that email checking process, initially I thought it was a bit of myth, but I’m now convinced that I work through my emails, more efficiently, quicker than I do when I’m sitting down.

Participant 16 (Group 1), electric desk, teacher/manager role

I think you do associate sitting with relaxing where as when I’m standing I am definitely more alert and far more productive.

Participant 1 (Group 2), electric, support/administration role

Equally, another staff member felt that standing was more of a distraction than facilitating her productivity:

Maybe you for sitting, maybe that impacts whether or not you use the desk or not, ‘cause I really can’t, I don’t feel focused at all when I’m standing. I’m shuffling from foot to foot and I’m usually just reading a document but the screen is too close or too it’s far away or my arms are too funny or whatever it might be. It’s just not comfortable…I don’t feel in the zone as it were.

Participant 15 (Group 2), electric desk, researcher role

Reflecting the quantitative findings reported above, the desk position felt to best serve productivity across the interviewees was not wholly determined by the particular task being performed as the following quotes show:

I think probably my usage has increased, so now I don’t really go to the low mode unless maybe some reading or some other activities which I find I just want to sit.

Participant 18 (Group 2), electric & manual desk, teacher/manager role

But also I guess typing it’s not a natural thing for me to stand and type I suppose. I’m happy reading so I’m almost the opposite to [Participant 18] really, happy reading, but I couldn’t type whilst I was there.

Participant 15 (Group 2), electric, researcher role

Interviewer: And what sort of tasks would you do standing?

All the tasks I do sitting, basically. emails, you know, writing documents, reading documents, phone calls, so everything.

Participant 11 (Group 3), manual desk, support/administrative role

A number of staff instead linked their preferences and productivity whilst sitting or standing to their habitual styles and past modes of working. For example, one felt he was more productive standing because he was previously in the military where decisions were literally made by thinking “on your feet”; another remarked he was \used to carrying out administrative tasks standing as he had worked in retail. Similarly, the employee quoted directly above attributed her better thinking when seated to her background as a student when she studied sitting down.

#### Desk/office set-up/context

Maintenance and extent of use of the desk in standing mode was also reportedly affected by the degree to which the desk set-up accommodated height adjustment.

And so the way that the desks were installed is that they’re down quite low so they’re at seated height but then as soon as you wind it up it hits the pin board and it won’t go any further, so we actually had to pull it out so we could get it up.

Participant 1 (Group 2), manual & electric desks, support/administration role

Yeah I think people, maybe people would actually wind them up if the cables went nicely up with the computer…[so] the screen doesn’t fly off the desk.

Participant 3 (Group 1), manual, support/administration role

Other issues related to the broader context of the office space:

…Unfortunately my desk has become a storage space in itself. So the usefulness of the desk is limited by, or is affected by the storage space that I have around me. .. so, for me it is some of the other furniture issues which affects the usefulness of the stand-up desk.

Participant 19 (Group 1), manual, researcher role

‘Cause I think some of the stand up desks, it’s a lot of stuff in people’s offices … aren’t oriented to that height. So unless you sort of have a holistic view and say, I actually reckon …I’ll pop all those things up or I’ll put my bookcase up and I’ll stack it from the half to the top … or my whiteboard is going to be at that standing level where I don’t have to crouch, then I think you’d probably get a better feel for whether you know that whole working at height thing rather than just standing behind a desk at height.

Participant 18 (Group 2), electric & manual desks, teacher/manager role

The majority of the participants felt that there was a need for getting instruction, not so much to operate the desk as this was straightforward, but how it may best be setup not to cause injury and support use of the height adjustability. The following sentiment was typical in response to a question on whether they received any instruction:

Absolutely nothing… We got nothing from the manufacturers, just like well here’s your desk. Well, it’s just there, so we had to set up we had to set up a computer, whatever way it was, so there may be a way of actually doing it properly, I don’t know.

Participant 16 (Group 1), electric desk, teacher/manager role

Although not totally precluding use of the desk, there was a sense that the more ambivalent user may be less likely to develop the habit of transitioning between sitting and standing when such obstructions arose. As one interviewee concluded:

So information like that would have been quite useful maybe would have let me certainly to set up my office so that I might have actually [used the desk] a bit more than I have.

Participant 15 (Group 2), electric desk, researcher role

### Electric versus manual

Most of the factors described above did not vary by whether the desk was manual or electric. However, often mentioned was the longer time (a few minutes according to the interviewees) to change the desk between sitting and standing height with the manual adjustment mechanism which was thought by a couple of participants to influence initiation, for example:

Some adopted much earlier than others but I’ve heard that it, because it takes a couple of minutes to actually wind the desk up and down for the manual one, that people obviously prefer to have the electric one.

Participant 21 (Group 3), manual desk, teacher/management role

But I share my office with someone who’s got a manual desk, she was equally interested in the standing but she hasn’t used it and I think it’s ‘cause it’s manual.

Participant 13 (Group 3), electric & manual desk, support/administration role

Further, maintenance and extent of use of the height adjustability was also reportedly influenced by desk type:

I think I’m probably standing a good six hours and in fact my laziness is probably working to my advantage because once I wound it up, I haven’t wound it back down.

Participant 1 (Group 2), manual & electric desks, support/administration role

Yeah, yeah, they do it a lot more up and down during the day if it’s electric. I would do mine at least twice a day so yeah and it’s so easy.

Key informant, electric & manual desks, management role

Having a manual desk may deter trialling the desk, prolong being in either the sitting or standing position or completely discourage standing; that is the number of transitions may be lower among users of a wind-up rather than electric desk.

The key informant, amongst others, agreed in hindsight that it may have been better to have installed only electrically operated desks to encourage use and not foster resentment (albeit mild).

So if you had to go through this whole process again is there something that you might do differently do you think?

Yeah we’d probably, probably push for the electric adjustment everywhere. Because it really is the icing on the cake, it really makes the benefit accessible to everybody.

Key informant, electric & manual desks, management role

## Discussion

This piece of formative research, to our knowledge, is the first to qualitatively describe the usability and acceptability of sit-stand desks. Installation of sit-stand desks throughout this medium sized organisation was well-received and resulted in a reduction in reported sitting time for a number of employees. After three months, the distribution of the self-reported proportion of time spent sitting at work had widened to span proportions lower than half the working day among those who took part in the evaluation study. However, acceptance was not universal as three participants reported using the desk in the standing position only infrequently and one had not tried it at all. Health impacts, both potential and experiential dominated employees’ explanations of their uptake and maintenance of use of the adjustable height facility. Other explanations referred to the office setup, whether the desk was manually or electrically adjustable and whether the person felt they worked more productively sitting or standing.

Employees’ initiation and continued use (or otherwise) of the standing option in this organisation could be characterized by a number of trajectories. One group committed to using the standing option prior to installation and were persistent and frequent users. Any barriers encountered were resolved, work-arounds instituted to enable continued use of the desks.

A second group also reported enjoying using the standing option but the discovery was more unexpected. Like those who were prior committed users, these newly committed users continued to stand regularly, even enthusiastically, but unlike the former were not necessarily convinced of the idea before trying the desks. Commitment arose out of perceived improved productivity and/or experience of a health benefit (either resolution of a health problem or increased energy) and/or a good fit with established work habits after experimentation.

A third, “uncommitted” group also experimented with using the desks in standing mode, but further use was undermined by difficulties encountered when transitioning or feeling ill at ease standing whilst working. Second-hand reports suggested a fourth group who were not interested in even trying the desk in the standing mode. However, as no one interviewed fitted this profile, this could not be confirmed and perhaps suggests bias in the sample of employees who were interviewed towards users rather than non-users of the standing option.

The data presented here confirm and extend previous research evaluating sit-stand desks and workplace interventions to reduce sitting. The reduction in mean sitting time after three months was comparable to that in a study where objective measurements were used [13] and despite the fact that intervention group in the earlier study were academic researchers in sedentary behaviour well acquainted with the health effects of sitting. The data also suggested the proximal effects of the desks may not be limited to merely more standing. As found by Alkhajah et al. (2012) using inclinometers, there was evidence in our qualitative data that the desks facilitated greater movement as well as standing [13].

Electric versus manual height adjustment reportedly affected the initiation and frequency of transitions among the current sample, with manual desk users remaining longer in one position or another, confirming earlier research based on objective data [21]. Given preliminary evidence that the frequency and timing of transitions may be important in preventing deleterious long term health effects [3,10] practitioners should consider the balance between the added cost and ease of height adjustment as promoting transitions may also avoid the potential short term impacts associated with excessive sitting or standing [20,28]. Further, sentiments expressed by the more infrequent users in the current sample who thought they may have benefitted from greater instruction on set-up mirror Wilk’s et al. (2006) finding that the company that had made the greatest investment in education and motivation had the highest use of the sit-stand desk [21].

New information also emerged from our analysis. Both the quantitative and qualitative data showed that employees practices and preferences did not follow a strong deterministic relationship between task and choice to sit or stand, perhaps with the exception of meetings. Such variation suggests that the desks are suitable for a range of office workers whose occupations involve different emphases in terms of tasks required to be performed. Our analysis also highlighted the importance of promoting the potential health benefits of sit-stand desks beyond musculoskeletal effects and if made part of pre-implementation process may motivate, some employees to at least try the height adjustment facility. Further, gearing the rest of the office to facilitate transitions our data suggest may prevent the less motivated from lapsing back to leaving their desk at sitting height. Our trajectories show that short term decisions based on what might seemingly be minor issues can affect long term use of the desk. Additional file 1 summarises specific practical considerations arising from our research (too detailed to be provided here) that practitioners and evaluation researchers may use in the development of future workplace interventions.

Based on the formative research described here, a number of hypotheses regarding implementation could be tested. For example, future research could examine whether emphasising the potential immediate and long-term health benefits before installation and/or providing instruction on the best way to set up a height adjustable desk and the surrounding workspace increase commitment and therefore use of the desk in the standing position. Further, it would be instructive to know whether using fittings attached to fixed-height desks which lift only computer screens and keyboards encounter the same consequences [13,29]. The degree of interaction between occupational role and use of the standing option deserves investigation as the current study was too small to explore a systematic relationship. More long term hypotheses could include whether advice on ergonomically sound set-up promotes safe use and mitigates reversion to sitting because of injury and how standing and transition frequency change over the longer (than three months) term.

Further research of sit-stand desks’ broader effects on productivity, absenteeism and medical costs among office workers are warranted. The current study and recent research [14] indicate that office workers subjectively perceive benefits of sit-stand desk use in relation to their productivity and past experimental research has generally shown at least no reduction in short term productivity [15,16,18]. Other research has demonstrated that well-planned workplace health interventions can reduce the modifiable risk factors with which sedentary behaviour is associated [3,30] and can represent a reasonable return on investment [30]. Larger scale and longer term studies in real world settings with reduction in sedentary behaviour as a primary outcome [11] could establish the generalizability, sustainability and cost effectiveness of sit-stand desks and provide a basis for the business case for implementing them.

## Strengths and limitations

Strengths of this study include the natural setting in that the intervention was not externally prompted and therefore represented a real world context; the whole of office approach whereby the desks were the employees’ own desks and not “hot desks” as in previous research [29]; the ability to compare manual and electrical height adjustment; a substantial follow-up period (three months); and the qualitative methodology allowing detailed exploration of quantitative findings.

One limitation was the small number of participants, although this was somewhat due to the natural attrition and absences of staff members in the workplace. Therefore while tests on changes in sitting time were statistically significant, the confidence intervals were wide reflecting substantial variation in the data and results should therefore be interpreted with caution. We did not offer those who did not participate in the group interviews to complete a follow-up survey questionnaire. However, this would have added only one extra participant as the other two non-responders at follow-up requested to be removed from the study as a whole. Further, the sample may have been biased towards users, or at least triallers of the desk, as only one participant had not used the standing option at all, and 13 did not participate. Measurements of sitting were also self-report rather than objective and therefore future quantitative findings could be strengthened with objective monitoring such as accelerometers [13,29] or by measuring cardiometabolic biomarkers [10,13] although the latter entails substantial participant burden particularly for longitudinal studies. The self-report sitting measures used have, however, demonstrated good validity and reliability [23,24].

## Conclusion

In conclusion this study suggests that sit-stand desks may provide a practical and acceptable means of reducing sedentary time among some office workers. The one-off cost but potential continuing benefit of the desks makes them a viable option for workplaces looking to provide employees with options to reduce sitting at work. Management staff in organisations planning to purchase sit-stand desks may facilitate committed use with practical assistance and information prior to and at the time of installation concerning the health benefits and desk and office setup that accommodates transitions between sitting and standing. Future research could evaluate similar installations in a variety of settings where excessive sitting is prevalent. Such data is invaluable to organisations planning to implement sit-stand desks, and supports the growing commitment to reducing sedentary behaviour in the interests of public health.

## Endnote

^a^One participant had her baseline data excluded from analysis by her own request.

## Competing interests

The authors declare that they have no competing interest.

## Authors’ contributions

AG conceived of the study, participated in its design and coordination, carried out the group interviews, analysed the qualitative and quantitative data and drafted the manuscript. JYC participated in the study design and coordination, carried out the group interviews and key informant interview, contributed to the analysis and to the writing of the manuscript. HvP participated in the study design and contributed to the writing of the manuscript. AB participated in the study design, assisted with recruitment of participants and contributed to the writing of the manuscript. All authors read and approved the final manuscript.

## Pre-publication history

The pre-publication history for this paper can be accessed here:

http://www.biomedcentral.com/1471-2458/13/365/prepub

## Supplementary Material

Additional file 1 Summary table of strategies and specific considerations for implementing sit-stand desks to reduce sitting in the workplace. Click here for file
