# Supplementary material for: “Thinking on your feet”: A qualitative evaluation of sit-stand desks in an Australian workplace
Source: BMC Public Health. 2013 Apr 18;13:365. doi: 10.1186/1471-2458-13-365 (PMC3643835; doi:10.1186/1471-2458-13-365)
Supplement: Additional file 1 — Summary table of strategies and specific considerations for implementing sit-stand desks to reduce sitting in the workplace. [file 1471-2458-13-365-S1.docx]

**Additional file 1:**  Summary table of strategies and specific considerations for implementing sit-stand desks to reduce sitting in the workplace

| **Aim** | **Strategy** | **Detail** |
| --- | --- | --- |
| **Promoting initiation** | Acquaint staff with potential health benefits of standing | - Musculoskeletal benefits - Prevention of chronic disease - Prevention of weight gain - Increased energy (evidence from subjective assessments only) - The importance of transitions between sitting and standing |
|  | Encourage experimentation adjusting the desk | - If implemented with other changes to office furniture, make desks salient - Use instructions on desk operation and office set-up as formal prompt for use |
| **Facilitating sit-stand transitions** | Provide worksurface that is easily adjusted | - Purchase electrically operated height adjusted desks or simple to operate desk-attachments |
|  | Assess and adjust surrounding furniture | - Ensure pin boards do not hinder adjustment of desk - Ensure sufficiently length of computer and telephone cables - Bundle cabling for computer - Consider adapting height of workstation partitions in shared office space - Ensure sufficient, easily accessible storage space around the desk |
| **Encouraging ergonomically safe use** | Provide adequate assistance/instruction on set-up and use | - Instructions should cover:   - Correct posture whilst sitting and standing   - Height of desk for safe use of computer keyboard and screen whilst standing   - Appropriate footwear |
| **Sustaining use** | Provide continuing support to users and non-users | - Allow for feedback on difficulties with use of height adjustment and address in a timely manner - Electronic reminders to transition between sitting and standing |
